# Statistical analysis plan for a cluster randomised controlled trial to compare screening, feedback and intervention for child anxiety problems to usual school practice: identifying Child Anxiety Through Schools-identification to intervention (iCATS-i2i)

**DOI:** 10.1186/s13063-023-07898-6

**Published:** 2024-01-17

**Authors:** Susan Ball, Tessa Reardon, Cathy Creswell, Lucy Taylor, Paul Brown, Tamsin Ford, Alastair Gray, Claire Hill, Bec Jasper, Michael Larkin, Ian Macdonald, Fran Morgan, Jack Pollard, Michelle Sancho, Falko F. Sniehotta, Susan H. Spence, Jason Stainer, Paul Stallard, Mara Violato, Obioha C. Ukoumunne

**Affiliations:** 1 National Institute for Health and Care Research (NIHR) Applied Research Collaboration (ARC) South West Peninsula (PenARC), Department of Health and Community Sciences, Faculty of Health and Life Sciences, University of Exeter, Exeter, UK; 2 Departments of Experimental Psychology and Psychiatry, University of Oxford, Oxford, UK; 3 Oxford NHS Foundation Trust, Oxford, UK; 4 Bransgore C of E Primary School, Christchurch, UK; 5 University of Cambridge and Cambridge and Peterborough Foundation Trust, Cambridge, UK; 6 Nuffield Department of Population Health, Health Economics Research Centre, University of Oxford, Oxford, UK; 7 School of Psychology & Clinical Language Sciences, University of Reading, Reading, UK; 8 Parents and Carers Together, Suffolk, UK; 9 Life and Health Sciences, Aston University, Birmingham, UK; 10 Charlie Waller Trust, Thatcham, UK; 11 Square Peg, East Sussex, Eastbourne, UK; 12 UK Health Security Agency, HCAI, Fungal, AMR, AMU and Sepsis Division, London, UK; 13 West Berkshire Council, Newbury, UK; 14 NIHR Policy Research Unit Behavioural Science, Newcastle University, Newcastle upon Tyne, UK; 15 Division of Public Health, Social and Preventive Medicine, Center for Preventive Medicine and Digital Health (CPD), Universitätsmedizin Mannheim, Heidelberg University, Heidelberg, Germany; 16 School of Applied Psychology and Australian Institute of Suicide Research and Prevention, Griffith University, Brisbane, Australia; 17 Stanley Primary School, Strathmore Road, London, UK; 18 Department of Health, University of Bath, Bath, UK

**Keywords:** Statistical analysis plan, Screening, School based, Anxiety problems, Cluster randomised controlled trial, Estimand

## Abstract

**Background:**

The Identifying Child Anxiety Through Schools-identification to intervention (iCATS-i2i) trial is being conducted to establish whether ‘screening and intervention’, consisting of usual school practice plus a pathway comprising screening, feedback and a brief parent-led online intervention (OSI: Online Support and Intervention for child anxiety), bring clinical and health economic benefits compared to usual school practice and assessment only — ‘usual school practice’, for children aged 8–9 years in the following: (1) the ‘target population’, who initially screen positive for anxiety problems according to a two-item parent-report child anxiety questionnaire — iCATS-2, and (2) the ‘total population’, comprising all children in participating classes. This article describes the detailed statistical analysis plan for the trial.

**Methods and design:**

iCATS-i2i is a definitive, superiority, pragmatic, school-based cluster randomised controlled trial (with internal pilot), with two parallel groups. Schools are randomised 1:1 to receive either screening and intervention or usual school practice. This article describes the following: trial objectives and outcomes; statistical analysis principles, including detailed estimand information necessary for aligning trial objectives, conduct, analyses and interpretation when there are different analysis populations and outcome measures to be considered; and planned main analyses, sensitivity and additional analyses.

**Trial registration:**

ClinicalTrials.gov ISRCTN76119074. Registered on 4 January 2022

**Supplementary Information:**

The online version contains supplementary material available at 10.1186/s13063-023-07898-6.

## Background

The iCATS programme comprises five work packages (WPs) outlined below. This article presents the statistical analysis plan (SAP) for WP5 — a school-based cluster randomised controlled trial (RCT) evaluating the pathway from universal screening to intervention for children with anxiety problems.

### WP1 establish an assessment system for universal screening of anxiety problems in children

WP1 was a psychometric study to develop a brief assessment system for child anxiety problems that is acceptable to children, parents/carers (henceforth referred to as parents) and teachers and to establish an algorithm and cut-offs that detect children with anxiety problems with high levels of sensitivity and specificity [1].

### WP2 develop the pathway from universal screening to brief intervention

WP2 involved working with stakeholders to develop pathway procedures and materials. The pathway was informed by existing knowledge from the empirical literature, from experiences of other trials of primary school-based interventions [2, 3], from codesign of the online support and intervention (OSI) programme [4, 5] and from relevant theory that has been used successfully within school settings (e.g. Normalisation Process Theory [6]).

### WP3 test the feasibility of the pathway

WP3 was a single-arm feasibility study to ensure the pathway is acceptable; no negative impacts/harms, trial recruitment and retention targets are feasible; and proposed clinical and economic measures are relevant and meaningful [7].

### WP4 model economic impact of elevated child anxiety

WP4 involves establishing the short-/medium-/long-term mental health outcomes and economic burden of elevated child anxiety and providing the parameters to estimate medium-term economic benefits/costs in the trial [8].

### WP5 evaluate the pathway from universal screening to brief intervention for children with anxiety problems

WP5 is a cluster RCT (with internal pilot) to establish whether usual school practice and the pathway developed in WP2 and tested in WP3 (comprising universal screening, feedback and treatment — *screening and intervention* arm) bring clinical and health economic benefits compared to usual school practice and assessment only (*usual school practice* arm), for children with anxiety problems (initially identified as experiencing anxiety problems from the two-item parent-report child anxiety questionnaire — iCATS-2, developed in WP1). The protocol for the iCATS-i2i cluster RCT was published in 2022 and included a brief overview of the statistical analyses [9]. The International Conference on Harmonisation guidelines state that primary statistical analyses should be pre-specified, to prevent data-driven choice of analyses and selective reporting of outcomes [10], and recent guidelines support early publication of SAPs that prospectively describe planned analyses of RCTs [11]. The SAP detailed in this article was finalised in July 2023, during the follow-up period of the trial. The proposed analyses and presentation of findings follow the Consolidated Standards of Reporting Trials (CONSORT) 2010 guidelines for reporting parallel group randomised trials [12] and the extension for cluster randomised trials [13].

## Methods and design

### Trial objectives

The primary objective of the iCATS-i2i cluster RCT is to compare the proportion of children who are not experiencing anxiety problems at 12 months post-randomisation (the primary outcome) among children who screened positive for anxiety problems according to the parent-report iCATS-2 at baseline (i.e. the target population), between those allocated to screening and intervention and those allocated to usual school practice.

Secondary objectives are as follows: (1) compare the proportion of children who are not experiencing anxiety problems at 4 and 24 months post-randomisation, in the target population and among all children in participating classes where the parent did not opt-out their child (i.e. the total population), between trial arms; (2) compare the proportion of children in the total population who are not experiencing anxiety problems at 12 months post-randomisation, between trial arms; (3) compare measures of anxiety, depression and behavioural problems at 4, 12 and 24 months post-randomisation, among children in the target and total populations, between trial arms; (4) compare school attendance and academic attainment up to the end of Year 6 (aged 10–11) for children in the target population, between trial arms; (5) evaluate acceptability and experiences of iCATS procedures for screening, feedback and intervention to inform an integrated process evaluation; and (6) assess child and parent quality of life, service use, time and costs associated with OSI delivery, short- and medium-term cost-effectiveness of iCATS procedures for identifying and supporting children with anxiety problems.

This article focuses on the analyses planned to address the primary objective and secondary objectives (1) to (4).

### Brief trial overview

iCATS-i2i is a two-arm, definitive, superiority, pragmatic, parallel group cluster RCT (with internal pilot) in which schools (clusters) are randomised 1:1 to receive either screening and intervention or usual school practice. Randomisation of clusters was necessary because a component of the intervention (a lesson on recognising and managing anxiety) is delivered to groups (classrooms) of children. Schools were randomised rather than classrooms to minimise the chance of contamination across trial arms. Schools were eligible to participate if they were mainstream primary or junior schools with at least two Year 4 classes. Schools with fewer than 40 children in Year 4 and/or with a Mental Health Support Team in place at the time of recruitment were excluded. In recruited schools, two or three classes are randomly selected to participate, and parents of children in these classes are invited to take part and to complete the iCATS-2. The items in the iCATS-2 assess the extent to which a child’s fears, worries or anxiety causes distress (*Do your child’s fears, worries or anxiety upset or distress your child?*) and interfere with family life (*Do your child’s fears, worries or anxiety make things difficult for your family as a whole?*). Parents rate each item on a 4-point Likert scale (0 — *no*, *not at all*; 1 — *yes*, *only a little*; 2 — *yes*, *quite a lot*; 3 — *yes*, *a great deal*), and responses are summed to produce a total score with possible range 0 to 6. Higher scores indicate a higher level of child anxiety problems. A score of three or more identifies children with anxiety problems with 76% sensitivity and 80% specificity [1]. Children who screen positive on this questionnaire (score ≥ 3) at baseline comprise the *target population*. All children in participating classes whose parents do not opt-out (regardless of whether they screen positive, screen negative or the iCATS-2 is not completed) belong to the wider *total population*, not of primary interest but on which exploratory analyses will be performed. Analyses of the total population are planned because it is believed that iCATS procedures, including an intervention system of two components — one designed for children who screen positive for anxiety problems and one for all children in the class regardless of screen result, may bring benefits for children beyond the target group. Specifically, there is a lesson on managing fears, worries and anxiety that is delivered to the whole class, and OSI is also available on an optional basis for families where the child does not screen positive. These broader data will allow exploration of the effects across the wider population on outcomes at follow-up. Data are collected at baseline (before randomisation), 4, 12 and 24 months post-randomisation. Full details of the trial background, rationale and design have been published [9].

### Intervention

The pathway involves screening, feedback and access to a therapist-supported parent-led online intervention (OSI) for child anxiety problems, as outlined below. Full details are provided in the published protocol [9].

#### Feedback for parents on screening outcomes

Parents who complete the baseline iCATS-2 and provide their contact details receive a feedback letter telling them whether their responses indicate their child may be (screen positive) or is unlikely to be (screen negative) experiencing anxiety problems and providing information about OSI. Where children screen positive, parents are contacted to arrange a feedback call with a children’s wellbeing practitioner (CWP) to discuss the screening outcome and be offered OSI. Where children screen negative, the feedback letter explains that OSI is available for all parents who feel their child may benefit, regardless of initial iCATS-2 responses, and parents are invited to contact the trial team if they wish to discuss the intervention.

#### Class lesson on recognising and managing anxiety

The research team and/or school staff deliver an interactive whole-class lesson (approximately 60 min) on recognising and managing fears, worries and anxiety for each participating class (total population).

#### Parent-led online intervention (OSI)

OSI comprises seven weekly modules (modules 0 to 6), including audio versions of text, videos and animations, interactive activities and inbuilt questionnaire measures. Each module (approximately 20 to 30 min) is supported by a telephone call (approximately 20 min) with a CWP once a week for 7 weeks and a follow-up review about 4 weeks after the intervention is completed (module 7). Modules teach parents cognitive behavioural strategies to apply in their child’s day-to-day life, including how to explore their child’s anxious thoughts, testing these thoughts by facing fears and problem-solving challenges. An optional app-based game for the child is available that is designed to help motivate the child to face their fears. Schools in both arms continue to provide any usual support to families, and families can continue to seek and/or receive any other additional support from schools and/or other service providers throughout the trial.

### Sampling of classes and randomisation of schools

A computer-based random sampling procedure, written in R software [14] by SB and implemented by an independent statistician otherwise not involved in the trial, was used to select Year 4 classes in recruited schools. If a recruited school had two or three eligible Year 4 classes, all these classes were selected to participate in the trial. If a school had more than three eligible Year 4 classes, three of these classes were randomly selected to participate.

Eighty-four schools (clusters) were recruited in total; 42 schools randomised to each trial arm. Schools were randomised in two separate cohorts: cohort 1 comprising schools in the internal pilot phase (28 schools recruited between October and December 2021 and randomised in March 2022) and cohort 2 comprising schools in the main trial (56 schools recruited between May and September 2022 and randomised in November 2022). Continuation of the trial beyond the pilot phase was approved by the Data Monitoring and Ethics Committee (DMEC) and Programme Steering Committee (PSC), who reviewed trial progress against pre-specified progression criteria, detailed in the protocol [9]. Allocation of schools was stratified by whether the percentage of children eligible for free school meals (FSM) in the school was above the national median (21.6%) for primary schools in 2020/2021 [15], using block randomisation with block sizes of 2 and 4. To balance on the number of children, the schools were sorted by the number of enrolled children in the target population, within each stratum (FSM category), before being allocated to trial arm. This approach, with blocking, was used to balance allocation on the potentially prognostic factor of FSM (deprivation) whilst ensuring a tight balance on the number of children allocated to each trial arm (which might not happen if school size was used as a stratification variable, categorised in some way). This method has been used in other cluster RCTs [16–18]. The programme for generating the randomisation sequence was written in R software [14] by SB and implemented by an independent statistician not otherwise involved in the trial. The independent statistician passed the allocations to the trial manager, who assigned schools to their trial arm.

### Flow of participants

**Fig. 1:**
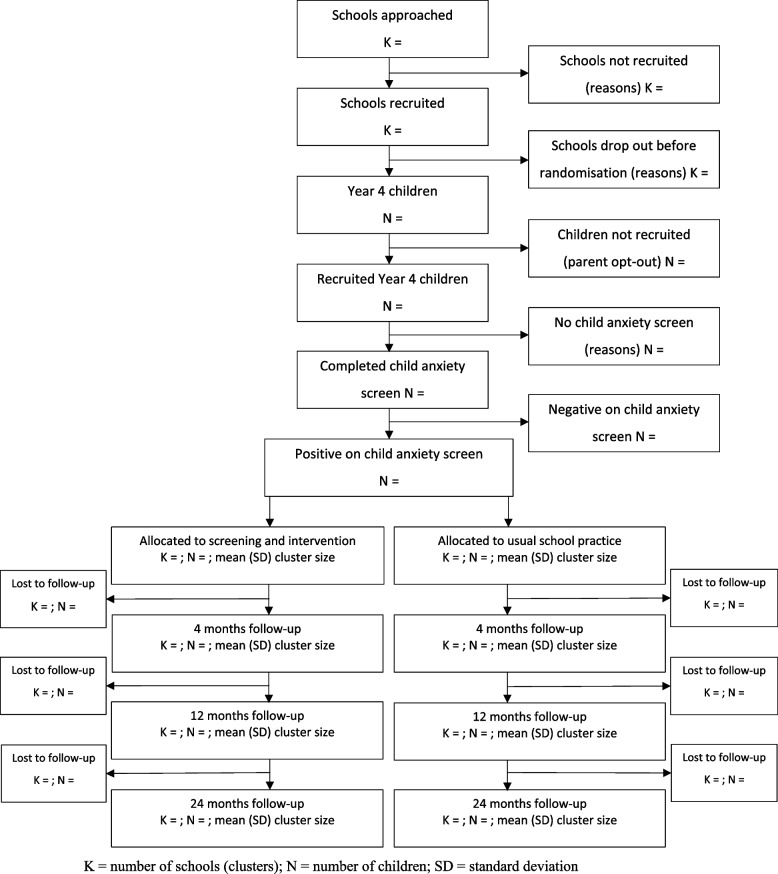
Flow of participants through the iCATS-i2i cluster randomised controlled trial — *target* population

**Fig. 2:**
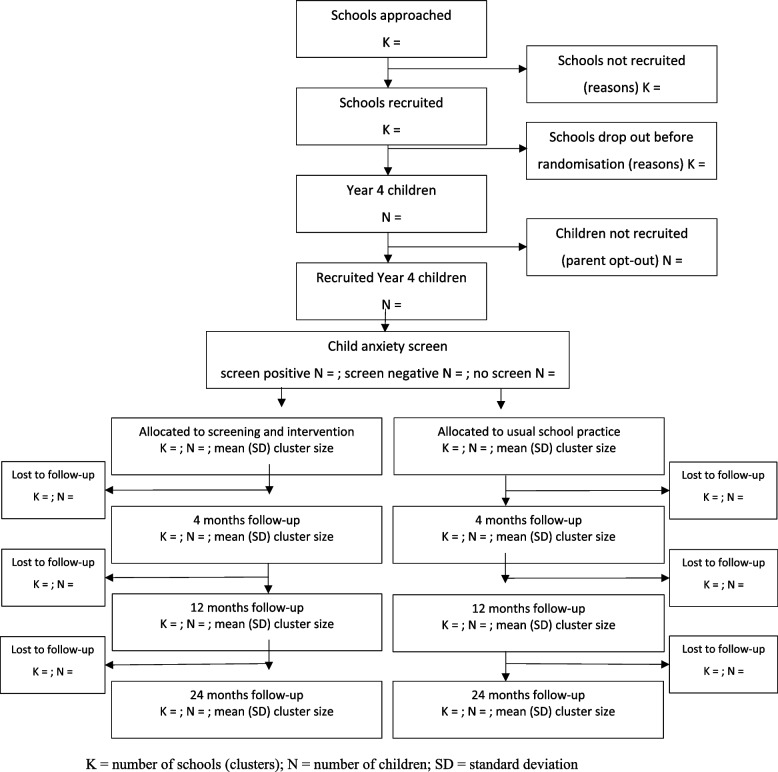
Flow of participants through the iCATS-i2i cluster randomised controlled trial — *total* population

The flow of participants through the trial will be reported in accordance with the CONSORT extension for cluster randomised trials (Figures 1 and 2) [13]. The flow diagrams will show, separately for the target and total populations, numbers of schools approached, recruited, and randomised and numbers of potentially eligible and eligible children. For the target population, numbers of children for whom a baseline parent-report iCATS-2 is completed, and who screen positive on the questionnaire, will be reported. For the total population, numbers of children who screen positive, screen negative, and do not have a completed baseline iCATS-2 will be reported. At each follow-up time point, numbers of schools and children and mean and standard deviation (SD) of cluster sizes will be reported.

The method of child selection for analyses in multiple-child families is described in Table 1.

**Table 1:** Method of child selection for analyses in multiple-child families

If there are two or more eligible children in a family, all children will be included in the study, and data will be collected on all of them. However, only one child will be selected to be included in the final analyses. This selection will use the following rules, which: (1) prioritise inclusion of children who screen positive and are, therefore, included in the target population and (2) ensure that, in the screening and intervention arm, the child who has been chosen (by their parent) to be the ‘target’ child for receiving the intervention is included in the analyses (in the target and total populations). • If only one child in the family screens positive on the baseline parent-report iCATS-2, this child will be included in the analyses (in the *target* population). • If more than one child in the family screens positive on the baseline parent-report iCATS-2, and the children are in the *usual school practice* arm, the child with the lowest study ID number out of those who screen positive will be included in the analyses (in the *target* population). • If more than one child in the family screens positive on the baseline parent-report iCATS-2, and the children are in the *screening and intervention* arm, the child who the parent has chosen to be the ‘target’ child for receiving the intervention, out of those who screen positive, will be included in the analyses (in the *target* population). If the parent does not choose any child to receive the intervention, the child with the lowest study ID number will be included in the analyses • If none of the children in the family screen positive on the baseline parent-report iCATS-2, and the children are in the *screening and intervention* arm, the child who the parent has chosen to be the ‘target’ child for receiving the intervention will be included in the analyses (in the *total* population). If the parent does not choose any child to receive the intervention, the child with the lowest study ID number will be included in the analyses. • If none of the children in the family screen positive on the baseline parent-report iCATS-2, and the children are in the *usual school practice* arm, the child with the lowest study ID number out of those who screen positive will be included in the analyses (in the *total* population).	

### Withdrawal/follow-up

Every effort is made to minimise withdrawal and loss to follow-up. Parents receive telephone, email and/or SMS reminders to complete follow-up measures, and regular update emails are sent. As well as reimbursement of any travel expenses, schools and families are offered payments for giving their time to participate in the trial.

School- (cluster) and participant-level loss to follow-up will be reported at each data collection time point, by trial arm and overall. A participant is deemed lost to follow-up at a given time point if the trial team are unable to facilitate any data collection (from any of the reporters for that participant) within the pre-specified window of +/− 4 weeks, but this will not preclude the participant providing data at later time points, if applicable. If a school withdraws from the trial during the follow-up period, the trial team continues to collect follow-up measures from children and parents. Where a child moves schools during the follow-up period, it is not possible to collect follow-up information and data from the child’s new school or class teacher, but the child remains in the trial and where possible the trial team continue to collect follow-up child- and parent-report measures (including the primary outcome). Where parents discontinue the online intervention, the trial team encourage them to stay in the trial and complete follow-up questionnaire measures.

### Outcomes

#### Primary outcome

The primary outcome is the proportion of children in the target population (initially identified as likely to benefit from the intervention based on scoring ≥ 3 out of 6 on the baseline parent-report iCATS-2) who score below the cutoff (i.e. score < 3) on the iCATS-2 12 months post-randomisation. Both items of the iCATS-2 must be completed for a total score to be obtained. This binary primary outcome reflects the aim to reduce anxiety so that it no longer interferes in daily life and, as such, would not require further intervention. The primary endpoint is 12 months because of the following: (i) it is not anticipated that beneficial effects will be delayed beyond 12 months, and (ii) effects will be important even if not maintained to 24 months.

#### Sample size

Sample size calculations were based on detecting an increase in remission of anxiety problems (with remission defined as scoring < 3 on the parent-report iCATS-2) from 50% in the usual school practice arm to 70% in the screening and intervention arm, with 90% power at the 5% level of significance. Seventy percent remission is similar to global treatment outcomes achieved beyond 6 months from brief, parent-led cognitive behavioural therapy in primary child and adolescent mental health settings [19, 20]. Fifty percent is a conservative (i.e. upper) estimate of natural remission among children at this age over 12 months based on data showing 50% remission in anxiety disorders in children in community settings over a 2-year period between 9 and 11 years of age [21]. Stakeholder consultation (including school staff and public health commissioners) indicated that an increase in remission of 20 percentage points would be considered worthwhile.

The original sample size requirement was 60 schools and 432 children in the target population (30 schools and 216 children in each trial arm), based on the following assumptions: two classes are sampled for participation from each recruited school; there are 30 children in each class; cluster sizes are equal (i.e. no allowance for variation in cluster size); 60% of parents complete the iCATS-2 for their children; 20% of children screen positive; 80% of recruited children are followed up; and the intra-cluster (intra-school) correlation coefficient (ICC) of the primary outcome is 0.05 [22].

The sample size calculation was updated, based on information from the feasibility study (WP3), which had smaller class sizes than originally assumed, a 21% screen positive rate for children and a 78% follow-up rate at 12 weeks. Given that the feasibility study coincided with substantial COVID-19-related restrictions and disruptions, it was reasonable to expect a modest improvement on recruitment/retention rates.

The updated sample size requirement was 80 schools and 398 children in the target population (40 schools and 199 children in each trial arm), based on the following assumptions: two or three classes (mean 2.3 classes) per school taking part, the mean number of children per class is 27, the coefficient of variation of the cluster size is 0.4 to compensate for unequal numbers of children followed up across schools, 40% of parents complete the iCATS-2, 20% of children screen positive, 80% of recruited children are followed up and the ICC of the primary outcome is 0.05 [22]. The design effect (variance inflation factor) is 1.18.

Further details of the sample size calculations are provided in the protocol [9].

#### Secondary outcomes

- *Two-item child anxiety questionnaire (iCATS-2)*: Parent-report, at 4, 12 and 24 months post-randomisation for the total population and at 4 and 24 months post-randomisation for the target population. The iCATS-2 will be analysed as a binary outcome for the target and total population. For the total population, this outcome will be analysed separately for the following: (i) all participants and (ii) those participants who screen negative.
- *Spence Children’s Anxiety Scale-8 item version (SCAS-8)*: Child-, teacher-, and parent-report, at 4, 12 and 24 months post-randomisation. An 8-item scale with each item scored 0 to 3 giving a total score 0 to 24. Higher scores indicate a higher level of child anxiety symptoms [23]. The score is scaled up pro-rata if at least 6 items are completed, by dividing the observed total score from the completed items by the maximum possible total score from the completed items and multiplying by 24. For example, if a total score of 12 is observed from 6 completed items, where the maximum possible total score from 6 items is 18, the score is scaled up to 16 (i.e. 12/18 × 24 = 16). The SCAS-8 will be analysed as a continuous outcome for the target and total population. For the total population, this outcome will be analysed for all participants.
- *Impact items*: Children and teachers complete the following items: child-report: *Do fears or worries upset you?*; *Do fears or worries stop you from doing things?*; and *Do your fears or worries make things difficult for people around you (e.g. family, friends, teachers)?* and teacher-report: *Do fears, worries or anxiety upset or distress this child?; Do this child’s fears, worries or anxiety make things difficult for you or the class as a whole?*. Responses to these items (where each item is scored 0 to 3, with the same response options as in the iCATS-2) are summed to provide total scores (child-report: range 0 to 9; teacher-report: range 0 to 6). Higher scores indicate a higher impact of child anxiety. All items must be completed for a total score to be obtained. These outcomes are collected at 4, 12 and 24 months post-randomisation and will be analysed as continuous variables for the target and total population. For the total population, these outcomes will be analysed for all participants.
- *Revised Children’s Anxiety and Depression Scale (RCADS)*: Child- and parent-report, at 4, 12 and 24 months post-randomisation. A 47-item scale with each item scored 0 to 3 [24, 25]. RCADS includes five anxiety subscales and one depression subscale. The *RCADS anxiety score* is calculated as the sum of the five anxiety subscale scores, including 37 items with a possible total score 0 to 111. Higher scores indicate a higher level of child anxiety symptoms. The *RCADS depression score* is calculated as the sum of the 10 items in the depression subscale, with a possible total score 0 to 30. Higher scores indicate a higher level of child depression symptoms. The total anxiety score can have up to 10 missing items but only if each of the five subscales has no more than two missing items; the total depression score can have up to two missing items. The scale total score is calculated as the sum of the completed items within that scale, divided by the number of items completed and multiplied by the total number of items in that scale; the result is rounded if necessary. For example, (1) if a child has an anxiety scale score of 52 from 27 completed items, with two missing items per subscale, the total score is calculated as follows: 52/27 × 37 = 71.3, rounded to 71; and (2) if a child has a depression scale score of 22 from 8 completed items, the total score is calculated as follows: 22/8 × 10 = 27.5, rounded to 28 [26]. RCADS anxiety and depression scores will be analysed as continuous outcomes for the target and total population. For the total population, these outcomes will be analysed for all participants.
- *Strengths and Difficulties Questionnaire (SDQ)*: Child- and parent-report, at 4, 12 and 24 months post-randomisation [27]. The full SDQ is collected for both reporters, at each time point. However, only the two subscales: *conduct problems* and *hyperactivity/inattention* will be analysed as outcomes. Both SDQ subscale scores are calculated as the sum of the five items in the subscale, with a possible total score 0 to 10 (each item is scored 0, 1 or 2; higher scores indicate higher levels of problems). Subscale scores are scaled up pro-rata if at least three items are completed, by dividing the observed total score from the completed items by the maximum possible total score from the completed items and multiplying by 10. For example, if a total score of 4 is observed from three completed items, where the maximum possible total score from three items is 6, the score is scaled up to 7 (i.e. 4/6 × 10 = 6.67, rounded to 7) [28]. These SDQ subscales will be analysed as continuous outcomes for the target and total population. For the total population, these outcomes will be analysed for all participants.
- Child Health Utility-9 Dimensions (CHU-9D) [29] and EQ-5D Youth version (EQ-5D-Y) instrument [30]: Child- and parent-report and EQ-5D five-level version (EQ-5D-5L) instrument [31]: parent self-report. These health-related quality-of-life outcomes are collected at baseline, 4, 12 and 24 months post-randomisation and will be analysed as part of the health economic analyses.
- *Learning related outcomes*: School attendance information provided by school staff at baseline, 4, 12 and 24 months post-randomisation. Subject to approval from the Department for Education and availability of data in time for main analyses of the trial, data on school attendance (years 4 to 6) and academic attainment (key stage 2 English and Maths national curriculum assessment outcomes) will be obtained from the National Pupil Database.

### General analyses principles

#### Participant population

Children who score ≥ 3 on the parent-report iCATS-2 at baseline are the primary sample of interest (target population), but the wider sample of all children in participating classes where the parent does not opt-out (total population) will also be followed up. Any children who score ≥ 3 on the iCATS-2 (i.e. screen positive) at baseline, but are not known to the trial team until after randomisation, will not be included in the target population but will be included in the total population.

Comparison of outcome data between the trial arms will use the intention-to-treat (ITT) principle with participants analysed according to the trial arm that their school was randomised to regardless of whether they received the intervention. The main analyses will be based on multiply imputed datasets.

No active data collection will take place outside the pre-specified data collection windows. However, if participants volunteer or request to provide data outside these windows, these will be collected and used in sensitivity analyses.

#### Estimand information

All estimands of interest will be based on participant-average effects. Full estimand information for the primary and secondary outcomes is given in Table 2.

**Table 2:** Estimand information for the primary and secondary outcomes in the iCATS-i2i cluster randomised controlled trial

Estimand aspect	Definition for different outcomes and analysis populations						
	Primary outcome: absence of anxiety problem at 12 months (target population)	Continuous secondary outcomes (target population)	Binary secondary outcomes (target population)	Continuous secondary outcomes (total population)	Binary secondary outcomes (total population)	Binary secondary outcomes (screen-negative population)	CACE analysis of primary outcome: absence of anxiety problem at 12 months (target population)
***Population***							
Schools	Mainstream primary or junior school with ≥ 2 Year 4 classes and > 40 Year 4 children, without a Mental Health Support Team in place at the time of recruitment						
Students	Children in participating classes who have anxiety problems (i.e. score ≥ 3 out of 6 on the parent-report iCATS-2 at baseline)			All children in participating classes whose parents do not opt them out		Children in participating classes who do not have anxiety problems (i.e. score < 3 out of 6 on the parent-report iCATS-2 at baseline)	Children in participating classes who have anxiety problems (i.e. score ≥ 3 out of 6 on the parent-report iCATS-2 at baseline)
***Treatment conditions***	Usual school care + the pathway (comprising universal screening, feedback and access to a therapist-supported parent-led online intervention) vs. usual school care + assessment for anxiety						
***Endpoint***	The absence of anxiety problems at 12 months (defined as a score < 3 on the parent-report iCATS-2)	Score on continuous measure at follow-up time point (4, 12, 24 months)	Classification according to outcome at follow-up time point (4, 12, 24 months)	Score on continuous measure at follow-up time point (4, 12, 24 months)		Classification according to outcome at follow-up time point (4, 12, 24 months)	The absence of anxiety problems at 12 months (defined as a score < 3 on the parent-report iCATS-2)
***Population-level summary measure***	Odds ratio	Mean difference	Odds ratio	Mean difference		Odds ratio	
***Participant- or cluster-average effect***	Participant-average						
***Handling intercurrent events***	Treatment policy (i.e. regardless of whether participants complete all modules, the treatment effect is described from the outcome measure in all participants)						Principal stratum (i.e. treatment effect is described for population of participants who would have completed at least modules 0–4)
Not completing all online modules							

*CACE*, complier average causal effect; *iCATS-2*, two-item parent-report child anxiety questionnaire

#### Levels of confidence and *p*-values

All hypothesis testing will be carried out at the (two sided) 5% level of significance. All between-group comparisons will be presented as the estimate with 95% confidence interval (CI) and *p*-value.

#### Unadjusted and adjusted analyses

Unadjusted and adjusted analyses will be carried out. Adjusted analyses will be considered the main analyses and will be adjusted for the following:

- Baseline value of the outcome, where collected. This will be a continuous variable for continuous outcomes and a categorical variable for binary outcomes. Specifically, the adjustment variable will have categories 3, 4, 5 and 6 for the primary (binary) outcome of the absence of anxiety problems in the target population; 0, 1, 2, 3, 4, 5 and 6 for the secondary outcome of absence of anxiety problems in the total population; and 0, 1 and 2 for the secondary outcome of the absence of anxiety problems in children who screen negative on the iCATS-2 at baseline.
- Child-level FSM status (binary variable)
- School-level FSM status (binary variable, the stratification variable)
- Cluster size (continuous variable, i.e. number of enrolled children in the target population, used in the randomisation process)
- Cohort status

Analysis of the iCATS-2 (binary outcome) in the total population will additionally be adjusted for the school-level percentage of children who screened positive on the questionnaire at baseline (out of those children for whom a questionnaire was completed).

#### Multiple testing

No adjustments will be made for multiple comparisons, and analyses of the secondary outcomes will be treated as exploratory.

#### Missing data

In instances where baseline data are not collected prior to randomisation, if these data are not outcomes, and are stable over time (e.g. school-level demographics and child-level demographics collected from schools), every effort is made to collect these retrospectively and they are considered baseline data. However, if these data are outcomes, and therefore not stable over time (e.g. teacher-report questionnaires on each child), data collection is not attempted after randomisation.

Separately for the target and total population and by trial arm and overall, the following will be reported:

- The percentage of missing observations for each outcome, at each follow-up time point
- Summary statistics for baseline characteristics, according to follow-up status at 12 months. Follow-up status is defined as children with data on at least one outcome, from at least one reporter, collected within the pre-specified data collection window versus children without any data on any outcomes, and from any reporters, collected within the data collection window.

Where there are missing items for outcomes with multiple items, the total score will be ‘scaled up’, based on rules for individual outcomes.

A joint modelling multiple imputation approach will be used, based on a multivariate linear mixed-effect model that accounts for clustering by including random effects at the school level. The imputation model will include all outcomes at all time points, health-related quality-of-life measures (from EQ-5D-Y, EQ-5D-5L, CHU-9D), trial arm status, cohort status, variables used to balance the randomisation and additional adjustments/prognostic factors.

The number of completed OSI modules will be used as an auxiliary variable, set to zero for participants in the usual school practice arm. Dummy (indicator) variables will facilitate inclusion of categorical variables, and imputation will follow the rule set out by Allison [32]. Values for imputation of continuous outcomes which fall outside the plausible range will *not* be rounded to within the plausible range, as this approach can introduce bias [33, 34]. The need for normalising transformations of variables pre-imputation will be considered.

Fifty imputed datasets will be generated in R software [14] using the packages *pan* and *mitml* [35]. If any problems are experienced in generating the imputed datasets, removal of non-essential variables from the imputation model will be considered.

The final statistical analyses will be carried out once all follow-up data have been collected, all data queries have been resolved and the database has been locked. Statistical analyses will be carried out using Stata version 17.0 or higher [36].

#### Data integrity

Details related to data processing, checking, cleaning and storage are specified in a study data management plan. Research Electronic Data Capture (REDCap) databases are used to capture data provided by participants via online surveys, and data collected on paper are manually entered by members of the research team. Data held in REDCap databases are stored on secure University of Oxford servers. Schools and participants are assigned unique IDs, and a document linking school/participant ID and personal details and contact information is stored separately from other data, with access restricted to members of the trial team involved in collecting data and delivering the intervention. At each assessment point, participants are asked to confirm current contact information and child’s current school, and records are updated where required.

#### Presentation of comparative analyses

For binary outcomes (including the primary outcome), results will be reported as the total number of children analysed in each trial arm, the number and percentage with the outcome of interest in each arm, unadjusted odds ratio (OR) and adjusted OR with 95% CI and *p*-value. Additionally, for the primary outcome at 12 months post-randomisation, an unadjusted risk difference with 95% CI will be obtained by specifying the identity link function instead of the logit link. For continuous outcomes, results will be reported as the total number of children analysed in each trial arm, the mean and SD of the outcome of interest in each arm, unadjusted mean difference and adjusted mean difference with 95% CI and *p*-value. ICCs from unadjusted analyses will be reported for all outcomes. A provisional table showing how the results from the main analyses of the primary outcome and secondary outcomes at 12 months will be presented is provided in the supplementary material.

### Proposed analyses

#### Baseline

Characteristics of schools, families and children (target and total populations) will be summarised by trial arm status and overall, using means and SDs (or medians and interquartile ranges) for continuous variables and numbers and percentages for categorical variables. Baseline measures of parent-report iCATS-2, child-, parent- and teacher-report child anxiety symptoms (SCAS-8 and impact items) and child- and parent-report child anxiety symptoms (RCADS anxiety score), depression symptoms (RCADS depression score) and behavioural problems (SDQ conduct problems and hyperactivity/inattention subscales) will also be summarised. No formal comparisons of baseline characteristics will be made between allocated trial arms, as recommended in the CONSORT 2010 guidelines [12].

#### Planned main analysis of the primary outcome

The primary outcome will be compared between trial arms using marginal logistic regression models using generalised estimating equations (GEEs) [37] with information sandwich (‘robust’) standard errors (SEs), assuming an exchangeable correlation structure. An assumption of the GEE model with exchangeable correlation structure is that the outcome and treatment effect are not related to the size of each cluster. This assumption is expected to hold in this trial. Explicitly, it is not anticipated that the number of children recruited from each cluster will be related to either the outcomes at follow-up or the effect of the intervention [38].

#### Planned analyses of the secondary outcomes

Binary secondary outcomes will be analysed using GEEs, as described for the primary outcome. Continuous secondary outcomes will be compared using mixed-effects linear regression, using the restricted maximum likelihood approach, including random effects at the school level to allow for the correlation between observations from the same cluster.

#### Planned additional analyses of the primary and secondary outcomes

The following sensitivity analyses will be carried out:

- Analysis of the primary outcome, using GEEs with an independent correlation structure, to check the assumption that the number of children recruited from each school is unrelated to either the outcomes at follow-up or the effect of the intervention
- Complete case analysis of the primary outcome and each of the secondary outcomes
- Analysis of the primary outcome and each of the secondary outcomes, including outcome data collected outside the pre-specified data collection windows
- Analysis of the primary outcome under the assumption that missing outcome data in the screening and intervention arm are missing not at random (MNAR), using a tipping point approach [39]. For the MNAR mechanism, it will be assumed that, in the screening and intervention arm, the prevalence of the primary outcome differs between children with a missing outcome and children with an observed outcome. To do the analysis, the following steps will be implemented following the multiple imputation process that assumes data are missing at random (as described above):

1. Calculate the proportion of children with the outcome (i.e. absence of anxiety problems at 12 months post-randomisation, according to the parent-report iCATS-2) in the screening and intervention arm, among those for whom the outcome was imputed. This is the *base* proportion.
2. Set the *missing parameter p* to take a range of values above and below the base proportion. These values of the missing parameter correspond to plausible proportions of children in the screening and intervention arm, for whom the outcome is missing, who could truly have the outcome.
3. For each value *p* of the missing parameter, simulate, using the Bernoulli model, the binary outcome (0 = anxiety problems, 1 = absence of anxiety problems) in the multiply imputed dataset for those in the screening and intervention arm with a missing outcome, with probability *p* of having the outcome.
4. In the same way as for the main analysis of the primary outcome, which uses the original multiply imputed screening and intervention arm outcomes, for each set of simulated outcomes from step 3, obtain an estimated OR and 95% CI.

By varying the values of the missing parameter, the *tipping point* can be identified. This is the point at which the general conclusion of the main analysis changes. The tipping point will be determined in one of two ways, depending on whether the main analysis of the primary outcome is statistically significant:

Option 1: the main analysis of the primary outcome is statistically significant. If the CI in the main analysis does not contain one for the OR (i.e. one trial arm is superior to the other), then the value of *p* at which the CI crosses one will be identified (the tipping point).

Option 2: the main analysis of the primary outcome is not statistically significant. If the CI in the primary analysis contains one for the OR (i.e. inconclusive whether one trial arm is superior to the other), then the value of *p* at which the CI no longer contains one will be identified (the tipping point).

The following additional analysis will be carried out:

- A complier average causal effect (CACE) analysis of the primary outcome. A participant will be classified as a complier if they complete at least the first five online modules (modules 0–4). The CACE analysis will provide an estimate of the intervention effect in the population of those that comply with the intervention (in contrast to the ITT analysis, which estimates the effect of randomisation in the full population). CACE analysis compares those who comply in the screening and intervention arm with those in the usual school practice arm who *would have* complied had they been offered the intervention.

A two-stage least squares (2SLS) instrumental variable approach will be used, extended from an existing approach [40] to also account for clustering using mixed-effects models at stage 1 and marginal models using GEEs at stage 2. At stage 1, compliance status will be regressed on trial arm allocation using mixed-effects linear regression, including a school-level random effect to account for clustering in compliance. At stage 2, a GEE including adjustments as specified for the main analyses, and an additional adjustment for the total of the residuals (at the school and individual level) obtained in stage 1 and a covariate for the compliance status, with robust estimates of SEs (specifying an exchangeable correlation structure), will be used to estimate the CACE. SEs for the CACE estimate will be obtained using cluster-level bootstrapping of the 2SLS procedure using the *cluster* option in Stata’s *bootstrap* command. Additional predictors of compliance may be considered for inclusion in stage 1 of the 2SLS procedure. Rubin’s rules will be used to estimate the SE of the CACE estimate across the imputed datasets.

### Harms

Any potential adverse events (AEs) will be recorded and managed in accordance with the trial AE protocol. Potential AEs will be recorded, logged and monitored by the principal investigator and iCATS management group. Serious AEs (SAEs) will be reported to the PSC and DMEC. In line with recent recommendations [41], a summary of all recorded AEs, and SAEs will be presented separately for the target and total population, by trial arm and overall. Planned analyses relating to positive and negative experiences of iCATS procedures for screening, feedback and intervention will be undertaken as part of a separate process evaluation. This will include analysis of qualitative interview data and quantitative data collected via a bespoke questionnaire (child, parent, teacher-report) to assess the acceptability of study procedures, including items specifically designed to assess negative experiences (e.g. ‘Taking part in the study was harmful for me and/or my child’).

## Discussion

The article reporting the protocol for this cluster RCT included a brief outline of the planned statistical analyses, which were subsequently further developed and modified after the start of the trial. This SAP was written and finalised during the follow-up period of the iCATS-i2i trial.

By publishing our detailed SAP for a school-based cluster RCT, we hope that it may be of use to other teams developing plans for similar trials, with similar considerations to be made. Presenting tabulated estimand information for this trial provides a way of summarising, in one place, necessary details for aligning trial objectives, conduct, analyses and interpretation when there are different analysis populations and outcome measures to be considered.

### Supplementary Information

**Additional file 1: Table 1.** Provisional table showing how the results from the main analyses of the primary outcome and secondary outcomes at 12 months will be presented. Main comparisons of outcomes in the target population at 12 months post-randomisation. Imputed data.

## Data Availability

Datasets and study materials generated during the current study will be made available in a public repository.

## Abbreviations

2SLS: Two-stage least squares
AE: Adverse event
CACE: Complier average causal effect
CHU-9D: Child health utility-9 dimensions
CI: Confidence interval
CONSORT: Consolidated standards of reporting trials
CWP: Children’s wellbeing practitioner
DMEC: Data monitoring and ethics committee
EQ-5D-5L: EQ-5D five-level version instrument
EQ-5D-Y: EQ-5D youth version instrument
FSM: Free school meals
GEE: Generalised estimating equation
iCATS: Identifying child anxiety through schools
iCATS-2: Two-item parent-report child anxiety questionnaire
iCATS-i2i: Identifying child anxiety through schools-identification to intervention
ICC: Intra-cluster correlation coefficient
ITT: Intention to treat
MNAR: Missing not at random
OR: Odds ratio
OSI: Online support and intervention (for child anxiety)
PSC: Programme steering committee
RCADS: Revised children’s anxiety and depression scale
RCT: Randomised controlled trial
REDCap: Research electronic data capture
SAP: Statistical analysis plan
SCAS-8: Spence children’s anxiety scale-8 item version
SD: Standard deviation
SDQ: Strengths and difficulties questionnaire
SAE: Serious adverse event
SE: Standard error
SMS: Short message service
WP: Work package
