# Supplementary material for: Statistical analysis plan for a cluster randomised controlled trial to compare screening, feedback and intervention for child anxiety problems to usual school practice: identifying Child Anxiety Through Schools-identification to intervention (iCATS-i2i)
Source: Trials. 2024 Jan 17;25:62. doi: 10.1186/s13063-023-07898-6 (PMC10795300; doi:10.1186/s13063-023-07898-6)
Supplement: Supplementary file 1 — Additional file 1: Table 1. Provisional table showing how the results from the main analyses of the primary outcome and secondary outcomes at 12 months will be presented. Main comparisons of outcomes in the target population at 12 months post-randomisation. Imputed data. [file 13063_2023_7898_MOESM1_ESM.docx]

**Supplementary material:** *provisional table showing how the results from the main analyses of the primary outcome and secondary outcomes at 12 months will be presented*

Main comparisons of outcomes in the **target** population at 12 months post-randomisation. Imputed data

|  | **Intervention arm (I)** | | **Control arm (C)** | | **Unadjusted** | **Adjusted mean difference (I-C) / odds ratio (I/C)^a^** | | | **ICC^b^** |
| --- | --- | --- | --- | --- | --- | --- | --- | --- | --- |
|  | **N** | **mean (SD) /**  **n (%)** | **N** | **mean (SD) /**  **n (%)** | **mean diff. (I-C) /**  **odds ratio (I/C)^a^** | **estimate** | **95% CI** | **p-value** |  |
| ***Primary outcome*** |  | |  | |  |  |  |  |  |
| Absence of child anxiety problems^†^ – Parent-report | * | * (*) | * | * (*) |  | * | * to * | * | * |
| ***Secondary outcomes*** |  |  |  |  |  |  |  |  |  |
| Anxiety symptoms | * | * (*) | * | * (*) |  | * | * to * | * | * |
| SCAS-8 – Child-report | * | * (*) | * | * (*) |  | * | * to * | * | * |
| SCAS-8 – Parent-report | * | * (*) | * | * (*) |  | * | * to * | * | * |
| SCAS-8 – Teacher-report | * | * (*) | * | * (*) |  | * | * to * | * | * |
| RCADS Anxiety – Child-report | * | * (*) | * | * (*) |  | * | * to * | * | * |
| RCADS Anxiety – Parent-report | * | * (*) | * | * (*) |  | * | * to * | * | * |
| Anxiety-related impact |  |  |  |  |  |  |  |  |  |
| Impact items – Child-report | * | * (*) | * | * (*) |  | * | * to * | * | * |
| Impact items – Teacher-report | * | * (*) | * | * (*) |  | * | * to * | * | * |
| Depression symptoms |  |  |  |  |  |  |  |  |  |
| RCADS Depression – Child-report | * | * (*) | * | * (*) |  | * | * to * | * | * |
| RCADS Depression – Parent-report | * | * (*) | * | * (*) |  | * | * to * | * | * |
| Behavioural problems |  |  |  |  |  |  |  |  |  |
| SDQ conduct problems – Child-report | * | * (*) | * | * (*) |  | * | * to * | * | * |
| SDQ hyperactivity/inattention – Child-report | * | * (*) | * | * (*) |  | * | * to * | * | * |
| SDQ conduct problems – Parent-report | * | * (*) | * | * (*) |  | * | * to * | * | * |
| SDQ hyperactivity/inattention – Parent-report | * | * (*) | * | * (*) |  | * | * to * | * | * |

^†^ Score less than 3 on iCATS-2; ^a^ Mean difference reported for quantitative outcomes and odds ratio reported for binary outcomes; ^b^ Intra-cluster (intra-school) correlation coefficients (ICCs) from crude (unadjusted) analyses; SD = standard deviation; CI = confidence interval; SCAS-8 = Brief Spence Children’s Anxiety Scale; RCADS = Revised Children’s Anxiety and Depression Scale; SDQ = Strengths and Difficulties Questionnaire.
